# *Tabebuia impetiginosa*: A Comprehensive Review on Traditional Uses, Phytochemistry, and Immunopharmacological Properties

**DOI:** 10.3390/molecules25184294

**Published:** 2020-09-18

**Authors:** Jianmei Zhang, Stephanie Triseptya Hunto, Yoonyong Yang, Jongsung Lee, Jae Youl Cho

**Affiliations:** 1 Department of Integrative Biotechnology, and Biomedical Institute for Convergence at SKKU (BICS), Sungkyunkwan University, Suwon 16419, Korea; zhangjianmei1028@163.com (J.Z.); stephunto@gmail.com (S.T.H.); 2 Biological and Genetic Resources Assessment Division, National Institute of Biological Resources, Incheon 22689, Korea; tazemenia@korea.kr

**Keywords:** *Tabebuia impetiginosa*, Taheebo, traditional uses, immunopharmacology, immunological disorders

## Abstract

*Tabebuia impetiginosa*, a plant native to the Amazon rainforest and other parts of Latin America, is traditionally used for treating fever, malaria, bacterial and fungal infections, and skin diseases. Additionally, several categories of phytochemicals and extracts isolated from *T. impetiginosa* have been studied via various models and displayed pharmacological activities. This review aims to uncover and summarize the research concerning *T. impetiginosa*, particularly its traditional uses, phytochemistry, and immunopharmacological activity, as well as to provide guidance for future research. A comprehensive search of the published literature was conducted to locate original publications pertaining to *T. impetiginosa* up to June 2020. The main inquiry used the following keywords in various combinations in titles and abstracts: *T. impetiginosa*, Taheebo, traditional uses, phytochemistry, immunopharmacological, anti-inflammatory activity. Immunopharmacological activity described in this paper includes its anti-inflammatory, anti-allergic, anti-autoimmune, and anti-cancer properties. Particularly, *T. impetiginosa* has a strong effect on anti-inflammatory activity. This paper also describes the target pathway underlying how *T. impetiginosa* inhibits the inflammatory response. The need for further investigation to identify other pharmacological activities as well as the exact target proteins of *T. impetiginosa* was also highlighted. *T. impetiginosa* may provide a new strategy for prevention and treatment of many immunological disorders that foster extensive research to identify potential anti-inflammatory and immunomodulatory compounds and fractions as well as to explore the underlying mechanisms of this herb. Further scientific evidence is required for clinical trials on its immunopharmacological effects and safety.

## 1. Introduction

Historically, people have used natural products such as plants, animals, microorganisms, and other biological resources to assuage and cure diseases [1]. Many of the commercial drugs (such as atropine, teniposide, aescin, digoxin, silymarin, and so on) available today were initially developed from plants and other biological resources used in traditional medicines [2,3]. Therefore, knowledge of the traditional use of natural products plays a large role in drug discovery and development.

*Tabebuia impetiginosa* (Mart. Ex DC. Mattos) is a plant belonging to the family Bignoniaceae, which is mainly distributed in the Amazon rainforest and other tropical regions of Central and Latin America [4]. It is not only a decorative plant but also has high pharmaceutical value. *T. impetiginosa* has been used as a traditional medicine to treat various diseases and has antinociceptive, anti-edematogenic, antibiotic, and antidepressant effects [5,6,7]. Moreover, the inner bark of this tree can be made into poultice or concentrated tea to treat various skin inflammatory diseases [8]. Several categories of compounds have been isolated and identified from *T. impetiginosa,* principally quinones, flavonoids, naphthoquinones, and benzoic acids [9,10,11,12]. In recent years, many investigations have demonstrated that extracts or compounds isolated from *T. impetiginosa* reveal an extensive range of pharmacological activities such as anti-obesity, antifungal, anti-psoriatic, antioxidant, anti-inflammatory, and anti-cancer activities [4,7,13,14,15,16,17,18]. It is particularly prominent in immunopharmacology. Typically, the mechanism of anti-inflammatory activity of extract from the inner bark of *Tabebuia* was studied through a molecular biological approach. Nevertheless, the clinical applications of *T. impetiginosa* have been poorly researched, and there is a void of information on its mechanisms of action.

As far as we know, no review in the literature provides a comprehensive summary of *T. impetiginosa*. Thus, in an attempt to provide a basis for the in-depth exploration and clinical application of this plant, we reviewed the traditional medicinal uses, botany, immunopharmacology, phytochemistry, and ethnopharmacology of *T. impetiginosa,* in addition to perspectives and possible directions of future research. Furthermore, this review will be conducive to identifying the information gaps important for future research into *T. impetiginosa*.

## 2. Methodology

A scientific literature review regarding the traditional uses, phytochemistry, anti-inflammatory, anti-cancer, antioxidant, and anti-autoimmune disease activities of *T. impetiginosa* was performed using bibliographic databases. Keywords used for this study were ‘anti-inflammatory,’ ‘*T. impetiginosa*,’ ‘Taheebo,’ and other names synonymous with Taheebo. Research articles were chosen if they tested a compound isolated from *T. impetiginosa* and investigated the related pharmacological activity. Studies using in vitro assays included inhibition of nitric oxide, oxidative enzymes, or cytokines. In vivo trials were included if they used antigens to induce ear edema, arthritis, or colitis in inflammatory animal models. Websites, articles, and scientific papers were used as sources of information on historical uses, components, taxonomy, and morphology of *T. impetiginosa*. Other sites, including mapchart.net, were used to create a distribution map of *T. impetiginosa.*

## 3. Taxonomy and Botanical Traits

### 3.1. Taxonomy

*T. impetiginosa* is also known as *Handroanthus impetiginosus* (Mart. ex Dc.) Mattos as accepted on the website www.theplantlist.org [19]. There are 17 valid synonymous names for *Handroanthus impetiginosus* (Mart. ex Dc.) Mattos and one invalid synonymous name (*Tecoma impetiginosa* Mart.). Synonymous names for *Handroanthus impetiginosus* (Mart. ex DC.) Mattos within one confidence level are *T. ipe* var. *integra* (Sprague) Sandwith, *Tecoma avellanedae* var. *alba* Lillo, *Tecoma ipe* var*. integra* Sprague, *Tecoma ipe* var*. integrifolia* Hassl., and *Tecoma ipe f. leucotricha* Hassl. Synonymous names for *Handroanthus impetiginosus* (Mart. ex DC.) Mattos within three confidence levels are *Gelseminum avellanedae* (Lorentz ex Griseb.) Kuntze, *Handroanthus avellanedae* (Lorentz ex Griseb.) Mattos, *T. avellanedae* (Lorentz ex Griseb.), *T. dugandii* Standl., *T. impetiginosa* (Mart. ex DC.) Standl., *T. nicaraguensis* S.F. Blake, *T. palmeri* Rose, *T. schunkevigoi* D.R. Simpson, *Tecoma adenophylla* Bureau and K. Schum, *Tecoma avellanedae* (Lorentz ex Griseb.) Speg., *Tecoma impetiginosa* Mart. ex DC., and *Tecoma integra* (Sprague) Hassl (The Plant List, 2013) (Table 1). This paper will use the name *T. impetiginosa* (Mart. ex DC.) Standl and present it as *T. impetiginosa*. *T. impetiginosa* is a member of family Bignoniaceae, genus *Tabebuia*, and species *impetiginosa* as written in NCBI: txid429701. Its genus name is derived from a native language of Brazil, while the species name is derived from the Latin word impetigo, a common and highly contagious skin infection. It was so named because people believed that this plant could be used to treat impetigo [20].

### 3.2. Botanical Traits

*T. impetiginosa* is also known as pink trumpet tree or purple trumpet tree due to its flower color [21]. In English, it is called Ipe, Taheebo (ant wood), and purple tabebuia. In French, it is known as Poui, while its Spanish names include Lapacho negro, lapacho, and quebracho. In German, the common name is Lapachobaum, Trompetenbaum, and Feenkraut. In Portuguese, this plant is recognized as Pau d’arco (bow tree), Ipe-roxo (red thick bark), and Ipe [19,20]. *T. impetiginosa* is well known due to its conspicuous appearance. This deciduous species can grow to a height of 30 m and sheds its leaves during the dry season. The palmately compound and serrated leaves are green and arranged in opposite or subopposite pairs. The leaf shape is elliptic or oblong with pinnate or banchidodrome venation. Showy purple, dark pink, or pink flowers appear in spring. The calyx is campanulate to tubular with five lobes and is trumpet shaped. Its fruit is contained in a pod and is comprised of an elongated cylindrical capsule with thin seeds [21,22].

### 3.3. Distribution

**Figure 1:**
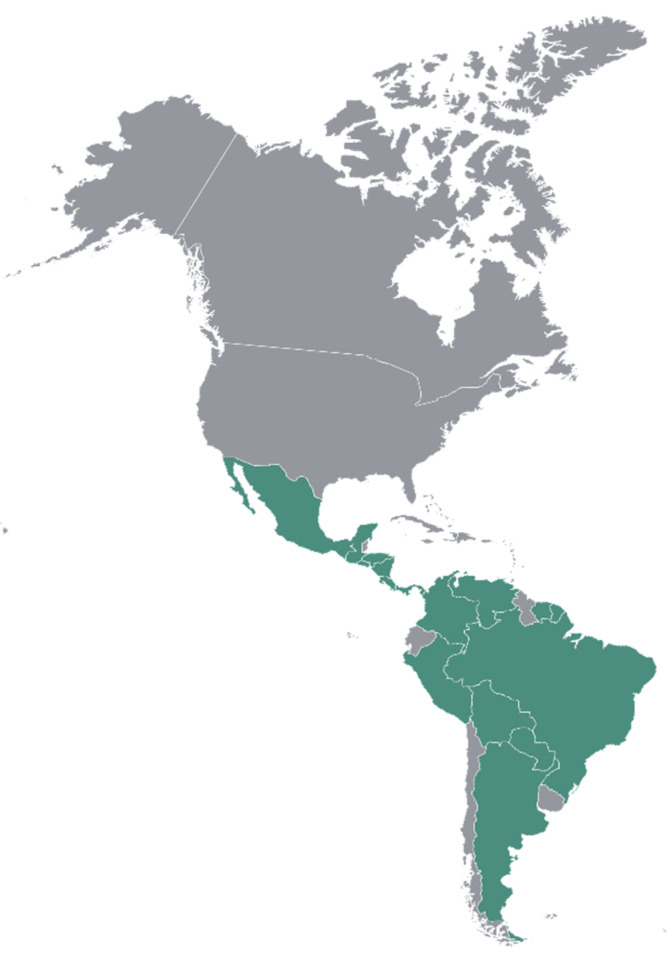
Distribution map of *Tabebuia impetiginosa.*

*T. impetiginosa* is a tree found in South and Central America and in some parts of North America. Information on the distribution of this species was obtained from www.tropicos.org [23] and a review paper on red lapacho [20]. Although it is best known for its presence in the Amazon rainforest, it is also found in Argentina, Bolivia, Brazil, Colombia, Costa Rica, Ecuador, El Salvador, French Guiana, Guatemala, Honduras, Mexico, Nicaragua, Panama, Paraguay, Peru, Suriname, Trinidad and Tobago, and Venezuela, as shown in Figure 1 and Table 2.

## 4. Traditional Uses

*T. impetiginosa* has been used traditionally to treat cancer [24], obesity [25], depression [26], viral, fungal, and bacterial infections [27], and inflammatory symptoms such as pain [28], arthritis [15], colitis [29], and prostatitis since the Inca civilization. The Callawaya Tribe makes a concentrated tea out of the tree’s inner bark for treating skin inflammatory diseases [8]. Moreover, it can be used as an astringent and diuretic [30]. Caribbean folk healers utilize the bark and leaves of *T. impetiginosa* to cure toothaches, backaches, and sexually transmitted diseases [31]. Latino and Haitian populations were also reported to use this plant for the treatment of infectious disease [32]. Brazilian people have traditionally used this plant for anti-inflammatory, analgesic, and antiophidic purposes against snake venom [33]. Traditional healers in Brazil prescribed *T. impetiginosa* for cancer and tumor prevention or treatment; 69.05% for the treatment of tumors and cancer in general and 30.95% for specific tumors or cancers [34]. Such ethnomedicinal uses of *T. impetiginosa* led us to pay attention to it for a full understanding of its immunopharmacological properties for the future development of an effective drug against ethnopharmacologically targeted diseases with this plant.

## 5. Phytochemistry

**Figure 2:**
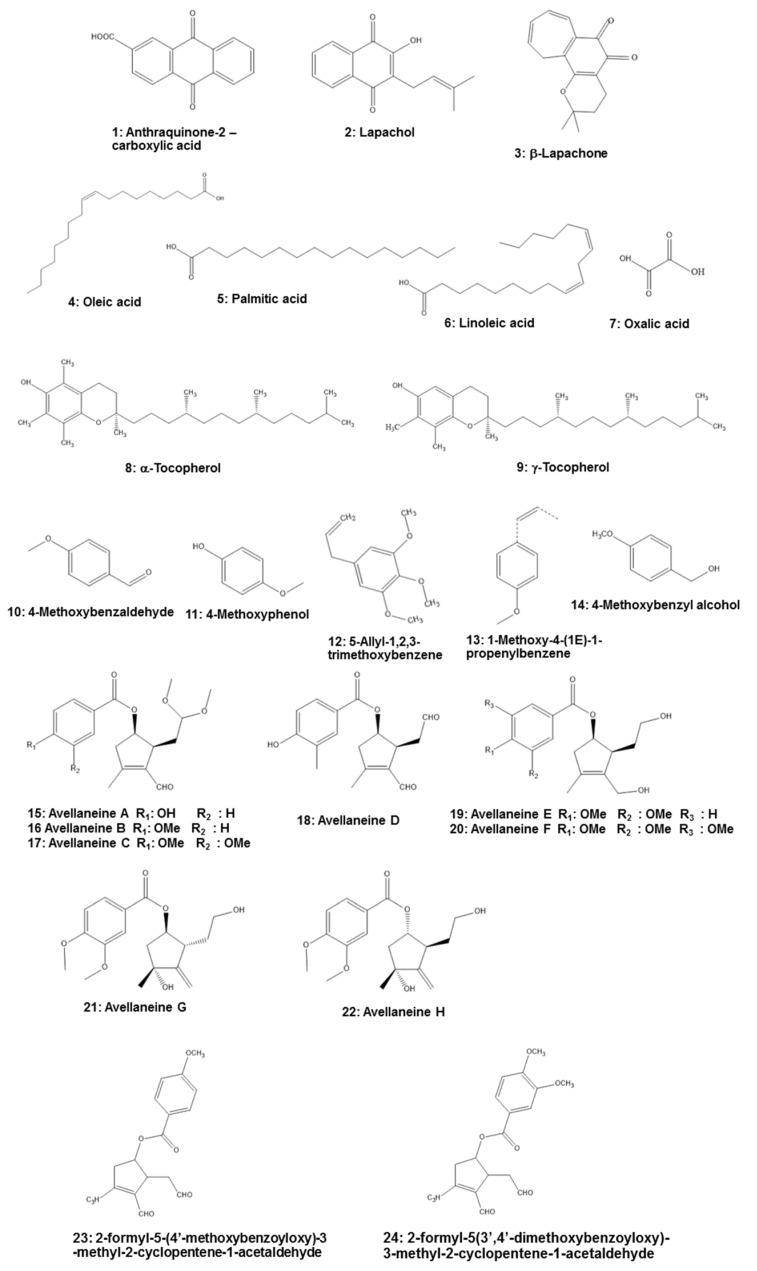
Chemical structures of *Tabebuia impetiginosa*-derived components.

Several categories of phytochemicals have been identified in the leaves, bark, and wood of *T. impetiginosa.* From *T. impetiginosa* bark, 19 glycosides comprised of four iridoid glycosides, two lignan glycosides, two isocoumarin glycosides, three phenylethanoid glycosides, and eight phenolic glycosides were methanol-extracted [35]. Major constituents of *T. impetiginosa* are furanonaphthoquinones, naphthoquinones, anthraquinones (e.g., anthraquinone-2-carboxylic acid (Compound **1** in Figure 2)), quinones, benzoic acid, flavonoids, cyclopentene dialdehydes, coumarins, iridoids, and phenolic glycosides [4,8,36]. The presence of naphthoquinones attracted scientific attention, with lapachol (**2**) and β-lapachone (**3**) especially piquing the interest of professionals in the medical field. Lapachol inhibits proliferation of tumor cells, while β-lapachone exhibits strong toxicity in murine and human cells. Lapachol has been shown to reduce the number of tumors caused by doxorubicin in *Drosophila melanogaster* heterozygous for the tumor suppressor gene. Lapachol can also decrease the invasion of HeLa cells, which could represent an interesting scaffold for the development of novel antimetastatic compounds [4].

Fatty acids, especially oleic acid (**4**), palmitic acid (**5**), and linoleic acid (**6**), are found in the bark of *T. impetiginosa*. Free sugars also were identified in the bark, with glucose being the most abundant, followed by fructose and sucrose. Organic acids, especially oxalic acid (**7**), are present, as well as the fat-soluble alcohols α-tocopherol (**8**) and γ-tocopherol (**9**). α-Tocopherol can reduce cardiovascular disease risk and neurodegenerative disorders [4]. In addition, *T. impetiginosa* has some volatile constituents that exhibit antioxidant activity. The major volatile constituents in *T. impetiginosa* include 4-methoxybenzaldehyde (**10**), 4-methoxyphenol (**11**), 5-allyl-1,2,3-trimethoxybenzene (**12**), 1-methoxy-4-(1*E*)-1-propenylbenzene (**13**), and 4-methoxybenzyl alcohol (**14**) [37].

Cyclopentene derivatives are secondary metabolites of plants, and this constituent from *T. impetiginosa* contained six known cyclopentenyl esters (avallaneine A–F (**15–20**)), two new cyclopentyl esters (avallaneine G (**21**) and H (**22**)), and two known cyclopentenyl esters. These cyclopentene derivatives may provide a significant anti-inflammatory effect on the lipopolysaccharide (LPS)-mediated inflammatory response by blocking the production of NO and PGE_2_; therefore, it is important to determine the molecular mechanism whereby cyclopentenyl esters from *T. impetiginosa* inhibit inflammatory responses [16]. Moreover, Koyama et al. [38] isolated two cyclopentene dialdehydes, 2-formyl-5-(4′-methoxybenzoyloxy)-3-methyl-2-cyclopentene-1-acetaldehyde (**23**) and 2-formyl-5-(3′,4′-dimethoxybenzoyloxy)-3-methyl-2-cyclopentene-1-acetaldehyde (**24**), that exert anti-inflammatory activity in human leukocytes. Thus, it is necessary to further investigate their activities.

## 6. Pharmacological Activities

Previous research has indicated various pharmacological effects of *T. impetiginosa* and its crude extracts and chemical compounds in a series of in vitro and animal models. It exhibits antibacterial, antioxidant, antifungal, antinociceptive, antidiabetic, anti-edema, anti-inflammatory, and anti-cancer activities at different concentrations or doses. The main pharmacological activities of extracts or compounds isolated from *T. impetiginosa* reported in in vitro and in vivo studies are briefly summarized in Table 3 and described in detail in the following subsections.

### 6.1. Anti-inflammatory Activity

#### 6.1.1. Regulation of Inflammatory Mediators.

*T. impetiginosa* can alter the expression of signaling molecules involved in the inflammation process, including nitric oxide (NO), prostaglandin (PGE_2_), and leukotriene B_4_ (LTB_4_) [8,15,28]. NO is essential for maintaining homeostasis and protecting human hosts and provides immunosuppressive effects as well as immunopathological and immunoprotective activities [44]. PGE_2_ is a mediator of inflammation, especially in diseases such as rheumatoid arthritis and osteoarthritis, playing an important role in stimulating the inflammatory response, facilitating tissue regeneration, and maintaining homeostasis [45]. LTB_4_, a pro-inflammatory lipid mediator, is synthesized from arachidonic acid, expressed on many inflammatory and immune cells, and is a powerful chemokine that promotes migration of macrophages and neutrophils to tissues [46]. *T. impetiginosa* can also inhibit the proinflammatory cytokines interleukin (IL)-1β and IL-6 [15,47]. IL-1β has an important homeostatic function; however, overproduction of IL-1β can result in pathophysiological changes related to pain and inflammation [48]. Likewise, IL-6 is a pro-inflammatory mediator with pleiotropic effects on immune response, inflammation, and hematopoiesis, but excessive production of IL-6 can cause various diseases [49]. In addition, the mRNA expressions of IL-1β and inflammatory genes inducible NO synthase (iNOS) and cyclooxygenase (COX)-2 were markedly decreased when treated with *T. impetiginosa* [8,15,28,47]. Information gained from in vitro and in vivo models provided insights to other researchers for further investigation. Some focused on macrophages, the main cells involved in inflammation, while others focused on neutrophils, the most abundant blood leukocytes, and their role in defense against pathogens [50,51]. Previously, Byeon et al. [8] discovered that suppression of PGE_2_ production negatively regulated the macrophage-mediated inflammatory response. Similarly, Suzuki et al. [52] found that *T. impetiginosa* repressed neutrophil activation. Interestingly, *T. impetiginosa* did not inhibit the migration of neutrophils but instead inhibited the reactive oxygen species (ROS) produced by migrating neutrophils. The ROS produced from normal cellular metabolism play an important role in the signaling pathways of plant and animal cells in response to environmental changes [53], and future studies should investigate their mechanisms and active substances in the context of neutrophil functional modulation.

Our body has two protective effects against infections in the form of innate and adaptive immune cells. Adaptive immune cells include T and B cells, while innate immune cells include macrophages, dendritic cells, and other cell types. Dendritic cells are the most effective antigen presenting cells due to their ability to express high levels of major histocompatibility complex II (MHC II), cluster differentiation 80 (CD80), and CD86 that are required for antigen presentation. This expression allows dendritic cells to effectively trigger an immune response [54]. Dendritic cells are predominantly found in two forms, mature and immature. Mature dendritic cells are important for stimulating the T cell immune response, while immature dendritic cells support T cell tolerance [55]. Previous research has discovered that the water extract of *T. impetiginosa* impacted dendritic cells by upregulating the expression of MHC II and CD86, the markers of dendritic cell maturation, but had no effect on production of pro-inflammatory cytokines. On the other hand, dendritic cells can affect the differentiation of CD4^+^ T cells, which are important for adaptive immunity, while treatment with *T. impetiginosa* can induce differentiation of CD4^+^ T cells, resulting in induction of Th2 and differentiation of regulatory T cells. The expansion of regulatory T and Th2 cells may suppress the Th1 response, thereby preventing dextran sulfate sodium (DSS)-induced colitis in mice [29].

#### 6.1.2. Effects on Inflammatory Signaling

When pattern recognition receptors (PRRs) interact with pathogen-associated molecular patterns (PAMPs) or damage-associated molecular patterns (DAMPs), intracellular signal transduction pathways are induced to activate and translocate transcription factors such as nuclear factor (NF)-κB, activator protein (AP)-1, signal transducer and activator of transcription 3 (STAT3), and interferon regulatory factor 3 (IRF3) into the nucleus to stimulate the expression of pro-inflammatory genes, thereby producing an inflammatory response [56]. NF-κB is one of the transcription factors that expresses pro-inflammatory genes and is involved in both innate and adaptive immune responses. NF-κB can be activated through canonical and non-canonical signaling pathways. The canonical NF-κB pathway is mostly involved in immune response, while the non-canonical NF-κB pathway is only involved in parts of the adaptive immune system [57].

Park et al. [15] used an immunoblotting technique to show that the ethanol extract of *T. impetiginosa* suppressed the activation of Src and spleen tyrosine kinase (Syk). Furthermore, to determine the direct molecular targets, they conducted kinase assays and found that both Syk and Src were suppressed by *T. impetiginosa* [46]. However, Byeon et al. [8] discovered that the water extract of *T. impetiginosa* did not function in the NF-κB pathway due to non-inhibition of phospho-IκB and the upstream molecules that activate phosphorylation of IκB and AKT. Results from Park et al. [15] showed inhibition of phospho-IκB, even though they did not assess AKT expression. However, phospho-Syk and phospho-Src upstream of AKT were inhibited by ethanol extracts of *T. impetiginosa*. These results could vary depending on the proportion of active components contained in extracts using different solvents [58].

Park et al. [59] investigated the effect of anthraquinone, a main component of *T. impetiginosa*. They specifically focused on anthraquinone-2-carboxlic acid (9,10-dihydro-9,10-dioxo-2-anthracenecarboxylic acid) (AQCA: **1**) and discovered through immunoblotting that an inhibitor of IκB (IKK) and IκBα decreased when treated with 100 μM of AQCA in LPS-induced RAW264.7 cells. They repeated the kinase assay and found that Syk and Src were inhibited by treatment with AQCA [59].

Another pathway, the mitogen-activated protein kinase (MAPK) pathway, activates the activator protein (AP)-1 transcription factor that can lead to expression of pro-inflammatory genes. The MAPK pathway consists of three families: extracellular-signal-regulated kinases (ERKs), c-Jun *N*-terminal kinases (JNKs)/stress-activated protein kinases (SAPKs), and p38s. ERKs can be divided into two subgroups: classic ERKs that include ERK1 and ERK2 and larger ERKs such as ERK5. Classic ERKs are mainly responsible for cell growth, survival, differentiation, and development. JNK family members, which include JNK1, JNK2, and JNK3, are stress-activated [60].

**Figure 3:**
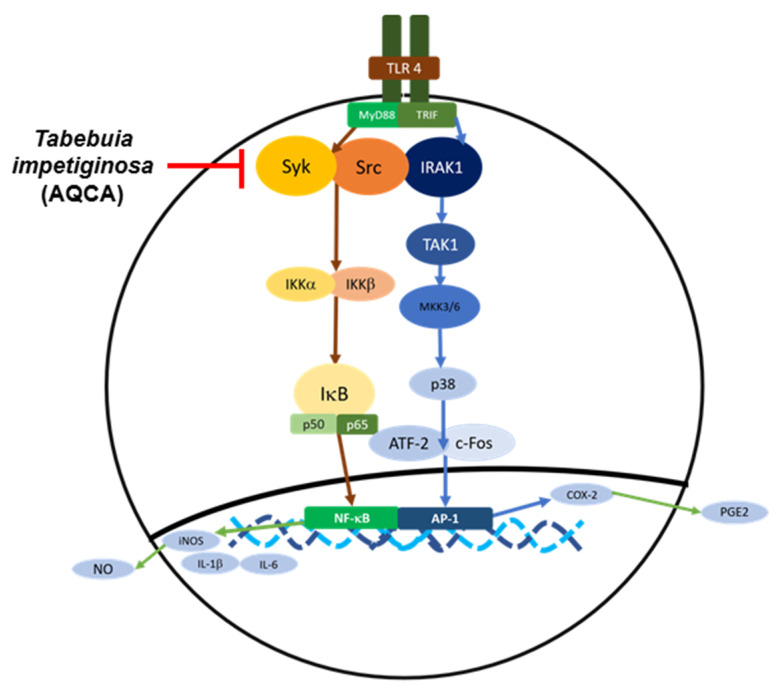
Inhibitory targets of *Tabebuia impetiginosa* in the NF-κB and AP-1 pathways.

Anthraquinone-2-carboxlic acid (AQCA) was identified as one of the major anthraquinones in *T. impetiginosa*. Administration of AQCA to mice treated with HCl/EtOH and aspirin resulted in reduced expression of phospo-p38 and interleukin 1 receptor associated kinase 1 (IRAK1) [61]. Treatment with AQCA reduced the expression of phospo-p38, c-JNK, mitogen-activated protein kinase 3/6 (MKK3/6), and transforming growth factor β-activated kinase (TAK1) in RAW264.7 cells. However, the expression of ERK was not inhibited. The upstream level of TAK1 was inhibited, as evidenced by degradation of IRAK1. These findings were confirmed using a conventional kinase assay with purified enzyme, and results showed potent suppression of IRAK1 by AQCA. Transfection was performed using HEK293 cells with the IRAK1 gene to validate the results, and treatment with AQCA suppressed phosphorylation of p38 protein without altering FLAG and IRAK1 protein levels. Taken together, these findings suggest that downregulation of IRAK1 by AQCA contributes to an anti-inflammatory effect [59]. Results are summarized in a pathway chart (Figure 3).

### 6.2. Anti-Cancer Activity

*T. impetiginosa* exhibits inhibitory effects on the growth of several human tumor cell lines, such as breast carcinoma (MCF-7), lung carcinoma (NCI-H460), cervical carcinoma (HeLa), and hepatocellular carcinoma (HepG2), and the GI_50_ values (corresponding to a sample concentration achieving 50% growth inhibition in human tumor cell lines) were 1.21, 1.03, 0.91, and 1.10 μg/mL, respectively [4]. Woo et al. [42] reported that β-lapachone isolated from *T. avellanedae* significantly inhibited the proliferation of human hepatoma cell line HepG2 by inducing apoptosis, which is associated with upregulation of pro-apoptotic Bax and downregulation of anti-apoptotic Bcl-2 and Bcl-X_L_ expression, proteolytic activation of caspase-3 and -9, as well as degradation of poly (ADP-ribose) polymerase protein.

In a human breast carcinoma derived estrogen receptor (ER^+^) MCF-7 cells model, Taheebo showed antiproliferative effects by upregulating xenobiotic metabolism-specific genes (dual specific phosphatase genes) and apoptosis-specific genes and by downregulating estrogen response and cell cycle regulatory genes [24]. Particularly, Taheebo treatment upregulated the dual specific phosphatase (DUSP) gene family and downregulated cyclin A and cdk2, indicating that Taheebo also inhibited the MAPK signaling pathway and phosphorylation of the ER *N*-terminal AF-1 domain [24]. Junior et al. [62] found that the anti-cancer activity of *T. impetiginosa* was correlated with the presence of lapachol and β-lapachone in its constitution. It is noteworthy that *T. impetiginosa* not only displayed growth inhibition against various tumor cell lines in vitro but also prolonged the duration of survival in a number of mouse models in vivo. For example, Queiroz et al. [63] examined the effects of *T. avellanedae* (30–500 mg/kg) and the naphtoquinone β-lapachone (1–5 mg/kg) in Ehrlich’s ascites tumor-bearing mice. They observed that *T. avellanedae* administration prolonged the lifespan of tumor-bearing mice by increasing the number of bone marrow granulocyte-macrophage colony-forming units and reducing colony-stimulating activity levels; the optimal biologically active dose was 120 mg/kg. In addition, Tahara et al. [14] found that naphthoquinones isolated from *T. avellanedae* markedly blocked the STAT3 pathway while reducing hyperactivation of these signals as well as inhibited growth of cancer cell lines.

### 6.3. Anti-Autoimmune Diseases

Recent research has shown that *T. impetiginosa* has effects on various autoimmune diseases such as psoriasis, osteoarthritis, allergy, and inflammatory bowel disease. Suo et al. [41] found that five novel compounds isolated from the water extract of *Taheebo* had strong anti-inflammatory activity but displayed weak or no effect on anti-allergic and antioxidant activities. Muller et al. [64] reported that Lapacho, a common constituent in the inner bark of *T. impetiginosa*, suppressed growth of the human keratinocyte cell line (HaCaT) and could be promising as an effective anti-psoriatic agent. In addition, it has been reported that *T. impetiginosa* bark extracts significantly inhibited the growth of some bacterial species associated with gastrointestinal disease and diarrhea, implying their suitability for prophylactic therapeutic usage [7]. Park et al. [15] examined the effect of *T. avellanedae* on monoiodoacetate-induced osteoarthritis in a Sprague-Dawley rat model. They observed that *T. avellanedae* administration ameliorated osteoarthritis symptoms by decreasing the serum levels of proinflammatory cytokines and inflammatory mediators, such as PGE_2_, LTB4, and IL-1β [15]. De Miranda et al. [5] further investigated its effects using animal models and described anti-edematogenic and antinociceptive effects of *T. impetiginosa* in rat paw edema induced by carrageenan. In this study, an aqueous extract containing a 200 mg/kg dose ameliorated rat paw edema in a way similar to indomethacin, the control drug. However, at a dose of 400 mg/kg, the edema was not reduced, suggesting that the edema-reducing compounds were competing with other constituents and nullifying any edema-reducing effect.

Lee et al. [28] investigated the analgesic and anti-inflammatory effects of *T. impetiginosa*, especially with regard to osteoarthritis. In this study, the analgesic effects were tested using pain threshold methods assessed by a hot plate test. A *T. impetiginosa* ethanol extract-treated group showed a significant analgesic effect at 200 mg/kg compared with a control group treated with diclofenac. Using an acetic acid-induced writhing response, they confirmed results from previous experiments that 100–400 mg/kg of *T. impetiginosa* ethanol extract significantly inhibited the number of writhes compared to the control group. This analgesic model used acetic acid because it causes inflammatory pain by increasing capillary permeability, and the hot plate-induced pain indicated narcotic involvement. Anti-inflammatory activity was assessed using acetic acid-induced vascular permeability, 12-*O*-tetradecanoylphorbol-13-acetate (TPA)-induced ear edema, arachidonic acid-induced mouse ear edema, and carrageenan-induced paw edema. Most of the group treated with *T. impetiginosa* exhibited reduced inflammation at a dose of 100–400 mg/kg, including suppression of ear weight and thickness, inhibition of ear inflammation, and reduction of edema in a TPA-induced ear edema test volume [28]. Byeon et al. [8] performed a similar study using a hot water extract and tested the edema model with different inducers. They found that prostaglandin E_2_ (PGE_2_) production was blocked and edema symptoms were reduced when treated with *T. impetiginosa*. However, in this study, *T. impetiginosa* only affected arachidonic acid-induced ear edema.

Park et al. [29] investigated the effect of *T. impetiginosa* on a DSS-induced colitis mouse model. They discovered that *T. impetiginosa* protected the colon from inflammation by reducing mucosal edema loss, epithelial crypts, and inflammatory cell infiltration. In addition, the use of *T. impetiginosa* in traditional arthritis medicines led researchers to perform experiments in an osteoarthritis model. Park et al. [46] used an ethanol extract of *T. impetiginosa* in the form of Tabetri™ (Ta-EE) in a monoiodoacetate-induced osteoarthritic mouse model. They compared the pain indicator of a mechanical paw withdrawal threshold to Von Frey stimuli and found that pain was significantly increased in osteoarthritic rats, which was suppressed by Ta-EE. Moreover, they also compared the results to those of methylsulfonylmethane (MSM) and Pc-LE and found that Ta-EE produced results comparable to those of these anti-inflammatory agents. Interestingly, results with Ta-EE were not dose-dependent, indicating that Ta-EE can be used in small doses. The osteoarthritic rats showed no weight loss, indicating no toxicity or side effects regarding weight loss or appetite caused by Ta-EE treatments. To investigate further, they measured the degradation of articular cartilage in rats administered Ta-EE and found it to be dramatically inhibited. Strangely, the chondroprotective effect of Ta-EE was better than that of MSM at 60 and 120 mg/kg doses in a dose-dependent manner.

## 7. Clinical Trials

In recent years, along with thorough research, some of the principal active components of *T. impetiginosa* have been used in clinical research. For example, β-lapachone, mainly distributed in heartwood of *T. impetiginosa,* has entered into phase 2 clinical trials for treatment of squamous cell carcinoma, and 2-acetylnaphtho (2,3-β) furan-4,9-dione, also referred to as STAT3 inhibitor BBI608 (Napabucacin), was developed by Boston Biomedical Inc [14].

## 8. Conclusions

In this paper, we summarized the traditional uses, botanical traits, phytochemistry, and pharmacological activities of *T. impetiginosa* with collation and analysis of relevant studies. *T. impetiginosa* has been used as a traditional medicine in Central and South America to treat edema, arthritis, diuretic, and infections. Based on its traditional use, in vivo and in vitro experiments examining its pharmacological potential have been conducted. In vivo experiments were conducted using edema, osteoarthritis, animal paw edema, and writhing (and other) models to screen effects of *T. impetiginosa*. Moreover, there are numerous studies confirming that extracts or compounds isolated from *T. impetiginosa* have various pharmacological activities such as anti-obesity, antibacterial, antifungal, antiviral, anti-psoriatic, antioxidant, anti-inflammatory, and anti-cancer activities.

Currently, substantial progress has been made in exploration of the phytochemistry and pharmacological activity of *T. impetiginosa.* Nonetheless, there are still challenges and gaps in published research papers that should be further explored to establish its clinical application value. Firstly, the extracts and compounds isolated from *T. impetiginosa* possess multiple pharmacological activities, though most functional mechanisms remain unclear and need to be further explored through in vivo and in vitro experiments. Furthermore, most studies on *T. impetiginosa* are still in the in vitro and in vivo mouse model stages. Toxicological research can be conducted on other animals such as rabbits in the future to evaluate its safety, which will pave the way for further clinical trials. In addition, further comprehensive experiments are needed to enrich the data and discover other pharmacological uses of *T. impetiginosa* and to find the exact mechanisms by which its extracts bind to target proteins.

## Figures and Tables

**Table 1:** Synonymous names for *Tabebuia impetiginosa* from The Plant List, 2013.

Synonym Name	Remarks
*Handroanthus impetiginosus* (Mart. ex Dc.) Mattos	Accepted name
*Tabebuia ipe* var. *integra* (Sprague) Sandwith	One confidence level
*Tecoma avellanedae* var. *alba* Lillo	One confidence level
*Tecoma ipe* var. *integra* Sprague	One confidence level
*Tecoma ipe* var. *integrifolia* Hassl.	One confidence level
*Tecoma ipe* f. *leucotricha* Hassl.	One confidence level
*Gelseminum avellanedae* (Lorentz ex Griseb.) Kuntze	Three confidence levels
*Handroanthus avellanedae* (Lorentz ex Griseb.) Mattos	Three confidence levels
*Tabebuia avellanedae* Lorentz ex Griseb.	Three confidence levels
*Tabebuia dugandii* Standl.	Three confidence levels
*Tabebuia impetiginosa* (Mart. ex DC.) Standl.	Three confidence levels
*Tabebuia nicaraguensis* S.F. Blake	Three confidence levels
*Tabebuia palmeri* Rose	Three confidence levels
*Tabebuia schunkevigoi* D.R. Simpson	Three confidence levels
*Tecoma adenophylla* Bureau and K. Schum	Three confidence levels
*Tecoma avellanedae* (Lorentz ex Griseb.) Speg.	Three confidence levels
*Tecoma impetiginosa* Mart. ex DC.	Three confidence levels
*Tecoma integra* (Sprague) Hassl	Three confidence levels
*Tecoma impetiginosa* Mart	Invalid name

**Table 2:** Geographical distribution of *Tabebuia impetiginosa.*

Species	Distribution
*Tabebuia avellanedae* Lorentz ex Griseb.	Argentina
*Handroanthus avellanedae* (Lorentz ex Griseb.) Mattos	Bolivia
*Bignonia heptaphylla* Vell.	Brazil
*Handroanthus impetiginosus* (Mart. ex DC.) Mattos	Bolivia, Brazil, Mexico
*Gelseminum avellanedae* (Lorentz ex Griseb.) Kuntze	Bolivia
*Tabebuia avellanedae* var. *paulensi*s Toledo	Brazil
*Tabebuia dugandii* Standl.	Colombia
*Tabebuia eximia* (Miq.) Sandwith	Bolivia, Panama
*Tabebuia heptaphylla* (Vell.) Toledo	Argentina, Bolivia, Brazil, Paraguay
*Tabebuia hypodictyon* (A. DC.) Standl.	Bolivia, Panama
* Tabebuia ipe (Mart.) Standl.	Panama
*Tabebuia ipe var. integra* (Sprague) Sandwith	Bolivia, Paraguay
*Tabebuia nicaraguensis* S.F. Blake	Nicaragua
*Tabebuia palmeri* Rose	Costa Rica, El Salvador, Guatemala, Honduras, Mexico, Nicaragua, Panama
*Tabebuia schunkevigoi* D.R. Simpson	Peru
*Tecoma adenophylla* Bureau ex K. Schum.	Brazil
*Tecoma avellanedae* (Lorentz ex Griseb.) Speg.	Honduras
*Tecoma avellanedae* var*. alba* Lillo	Argentina
*Tecoma eximia* Miq.	Brazil
*Tecoma hassleri* Sprague	Paraguay
! *Tecoma heptaphylla* (Vell.) Mart.	Panama
*Tecoma hypodictyon* A. DC.	Brazil
** Tecoma impetiginosa Mart.	Panama
!*Tecoma impetiginosa* Mart. ex DC.	Brazil
*Tecoma impetiginosa* var. *lepidota* Bureau	Brazil
*Tecoma integra (Sprague)* Chodat	Bolivia, Panama
*Tecoma ipe fo.* leucotricha Hassl.	Paraguay
** Tecoma ipe Mart.	Bolivia, Panama
*Tecoma ipe* var*. integra* Sprague	Paraguay
*Tecoma ipe* var*. integrifolia* Hassl.	Bolivia
*Tecoma ochracea* Cham.	Brazil

! = legitimate, * = illegitimate, ** = invalid.

**Table 3:** Immunopharmacological effects of *Tabebuia impetiginosa.*

Pharmacological Activity	Extract/Isolated Compounds	Model	Concentration/Dose	Results	Ref.
Immunomodulatory	Water extract	RAW264.7 (murine macrophage cell), U937 (human promonocytic cell)	50, 100, 200, and 400 μg/mL	Maintained cluster formation of RAW264.7 cells even after lipopolysaccharide (LPS) treatment. Downregulated the phagocytic uptake of FITC-labeled dextran. Upregulated cell-cell interactions by decreasing migration of cells and enhancing CD-29-mediated cell-cell adhesion and the surface levels of adhesion molecules and costimulatory molecules linked to macrophage stimulation, as seen in upregulation of reaction oxygen species (ROS) release. Suppressed an alteration in the membrane level of macrophages (phagocytic uptake and morphological changes).	[39]
	Ethanol extract	IL-2-independent T-lymphocyte	0.25, 0.5, 0.75, 0.9, and 1.0, mg/mL	Inhibited activation and proliferation of IL-2-independent T-lymphocyte	[40]
Anti-inflammatory	Water extract	LPS-stimulated macrophages, arachidonic acid, or croton oil-induced mouse ear edema models	0–400 μg/mL, 100–400 mg/kg	Inhibited the production of NO and PGE_2_ and suppressed the mRNA levels of COX-2 and iNOS. Curative effect in an in vivo PGE_2_-based inflammatory symptoms model induced by arachidonic acid.	[8]
	Ethanol extract	TPA- or arachidonic acid-induced ear edema, hot plate, acetic acid-induced vascular permeability in rats	100, 200, or 400 mg/kg	Inhibited inflammation of paw edema, ear inflammation, and dye leakage in the vasculature using various animal models including acetic acid-induced vascular permeability, 12-*O*-tetradecanoylphorbol-13-acetate (TPA)-induced ear edema, arachidonic acid-induced mouse ear edema, and carrageenan-induced paw.	[28]
	Five novel compounds	Human myeloma THP-1 cells	25 μM	Showed inhibitory activity on production of the inflammatory cytokines, such as TNF-α and IL-1β.	[41]
	Cyclopentene derivatives	RAW264.7 cells	12.5, 25, 50 μg/mL	Suppressed the production of NO and PGE_2_.	[16]
Anti-cancer	Naphthoquinones	MDA-BB-231, MCF7, and A549 cells	0–30 μM	Inhibited growth of cancer cell lines and STAT3 phosphorylation activity.	[14]
	Water extract	Estrogen receptor (ER)^+^ human mammary carcinoma MCF-7 cell line	0.05, 0.125, 0.25, 0.5, 0.75, 1.5 mg/mL	Exhibited dose-dependent growth inhibition of MCF-7 cells.	[24]
	β-lapachone	A549 human lung carcinoma cells		Inhibited growth of A549 cells and telomerase activity; induced apoptosis by reducing the expression of Bcl-2, increasing the expression of Bax, and activating caspase-3 and caspase-9.	[13]
	β-lapachone	HepG2 hepatoma cell line		Inhibited the activity of HepG2 by inducing apoptosis; downregulation of Bcl-2 and Bcl-X_L_, upregulation of Bax expression; induced apoptosis by activating caspase-3 and caspase-9 and degrading poly (ADP-ribose) polymerase protein.	[42]
	Methanol extract	Human tumor cell lines MCF-7, NCI-H460, HeLa, and HepG2; porcine liver primary cells (PLP2).	GI50 values: 110.76 ± 5.33 µg/mL (MCF-7), 76.67 ± 7.09 µg/mL (NCI-H460), 93.18 ± 1.46 µg/mL (HeLa), 83.61 ± 6.61 µg/mL (HepG2), and >400 µg/mL (PLP2).	Showed cytotoxic effects on MCF-7, NCI-H460, HeLa, and HepG2 cells.	[4]
Antinociceptive	Ethanol extract	Acetic acid-induced writhing response in rats	100, 200, or 400 mg/kg	Increased the pain threshold in a mouse model when assessed through the hot plate test and inhibited the number of writhes compared to controls in the acetic acid-induced writhing responses mouse model.	[28]
Osteoarthritis	Ethanol extract	RAW264.7 cells and chondrosarcoma cell line (SW1353); monoiodoacetate (MIA)-induced osteoarthritis in rats	75, 150, and 300 μg/mL	Showed a chondroprotective effect by preventing cartilage degradation through targeting of NF-κB and AP-1 signaling pathways in macrophage and chondrocyte cells. Downregulated MMP2, MMP9, and MMP13 in a PMA-induced, dose-dependent manner; no effect on the gene expression of COL2A1 and CHSY1.	[15]
Colitis	Water extract	RAW264.7 cells Dextran sulfate sodium (DSS)-induced colitis in mice	100, 300, 900, and 2700 μg/mL 2 mg/day, a total of 5 days	Activated DC to produce immunosuppressive IL10; upregulated anti-inflammatory Th2 and Foxp3^+^ Treg cells in mesenteric lymph node (MLN) and downregulated pro-inflammatory Th1 and Th17 cells. By upregulating type II T-assisted immune response, weight loss and inflammation of colon tissue were downregulated in DSS-induced colitis mice.	[29]
Antioxidant	Methanol extract		EC50 values: 0.68 ± 0.03 (DPPH scavenging activity), 0.27 ± 0.01 (Reducing power), 0.23 ± 0.04 (β-carotene bleaching inhibition), 0.14 ± 0.01 (thiobarbituric acid Thiobarbituric acid reactive substances (TBARS) inhibition).	Showed the highest antioxidant activity, which may be related to its total phenol content.	[4]
	Methanol, butanol, and water extracts	H_2_O_2_-induced NIH3T3 cells	0–2 mg/mL	Regenerated superoxide dismutase (SOD), catalase, and glucose 6-phosphate dehydrogenase activities; enhanced the concentration of glutathione in the cell; protected proteins from oxidative attack of H_2_O_2_, reduced formation of malondialdehyde in the cell, and protected NIH3T3 cells from H_2_O_2_-induced oxidative stress.	[43]
	Volatile constituents		5, 10, 50, 100, and 500 μg/mL	Displayed dose-dependent activity in antioxidant assays	[37]
	Phenylpropanoid glycosides		Compound 5 had the highest antioxidant activity, with an IC_50_ of 0.12 µM	Had inhibitory effects on cytochrome CYP3A4 enzyme	[18]
Anti-obesity	*n*-butanol extract	Ovariectomized (OVX) mice. 3T3-L1 cells	A total of 16 weeks	Preventing the accumulation of adipocyte in mice, weight loss and fat mass ↓ in ovariectomized mice.	[17]
	Ethanol extract	Triton WR-1339-treated Wistar rats	A total of 24,700 kJ/kg energy	Decreased postprandial triglycerides in rats given a fatty meal.	[25]
Anti-allergic	Five novel compounds	RBL-2H3 cells	100 μM	Inhibited release of β-hexosaminidase of the allergy marker.	[41]
Antidepressant	Ethanol extract	Forced swimming test (FST) and tail suspension test (TST) in mice.	100 mg/kg, p.o. (in the FST) and 10–300 mg/kg, p.o. (in the TST)	Produced antidepressant effects in the tail suspension test and forced swimming test.	[26]
Antiplatelet	Methanol extract	Rabbit platelets and cultured rat aortic vascular smooth muscle cells (VSMCs)	10, 50, 100, and 200 μg/mL	Reduced platelet aggregation by inhibiting arachidonic acid release and ERK1/2 MAPK activation.	[30]
